# Neurophysiological Correlates of Neuroanatomical Dimensions in Major Depressive Disorder: Electroencephalographic Markers of Brain Function and Treatment Outcome

**DOI:** 10.1016/j.bpsgos.2026.100750

**Published:** 2026-05-07

**Authors:** Wenyi Xiao, Rachel D. Woodham, Mathilde Antoniades, Dhivya Srinivasan, Yong Fan, Guray Erus, Jose A. Garcia, Stephen R. Arnott, Taolin Chen, Ki Sueng Choi, Cherise R. Chin Fatt, Benicio N. Frey, Faranak Farzan, Vibe G. Frøkjær, Melanie Ganz, Beata R. Godlewska, Stefanie Hassel, Keith Ho, Andrew M. McIntosh, Kun Qin, Susan Rotzinger, Matthew D. Sacchet, Jonathan Savitz, Haochang Shou, Ashish Singh, Aleks Stolicyn, Irina Strigo, Stephen C. Strother, Duygu Tosun, Dongtao Wei, Roland Zahn, Ian M. Anderson, W. Edward Craighead, J.F. William Deakin, Boadie W. Dunlop, Rebecca Elliott, Qiyong Gong, Ian H. Gotlib, Catherine J. Harmer, Sidney H. Kennedy, Gitte M. Knudsen, Helen S. Mayberg, Martin P. Paulus, Jiang Qiu, Madhukar H. Trivedi, Heather C. Whalley, Chao Gan Yan, Allan H. Young, Christos Davatzikos, Cynthia H.Y. Fu

**Affiliations:** a Department of Psychology, University of East London, London, United Kingdom; b Centre for Affective Disorders, Department of Psychological Medicine, Institute of Psychiatry, Psychology and Neuroscience, King’s College London, London, United Kingdom; c Center for Biomedical Image Computing and Analytics, Perelman School of Medicine, University of Pennsylvania, Philadelphia, Pennsylvania; d Indoc Research, Toronto, Ontario, Canada; e Department of Radiology, Huaxi MR Research Center, Institute of Radiology, Functional and Molecular Imaging Key Laboratory of Sichuan Province, West China Hospital of Sichuan University, Chengdu, Sichuan, China; f Xiamen Key Laboratory of Psychoradiology and Neuromodulation, Department of Radiology, West China Xiamen Hospital of Sichuan University, Xiamen, Fujian, China; g Nash Family Center for Advanced Circuit Therapeutics, Icahn School of Medicine at Mount Sinai, New York, New York; h Department of Psychiatry, Center for Depression Research and Clinical Care, University of Texas Southwestern Medical Center, Dallas, Texas; i Department of Psychiatry and Behavioural Neurosciences, McMaster University, Hamilton, Ontario, Canada; j Mood Disorders Treatment and Research Centre and Women’s Health Concerns Clinic, St. Joseph’s Healthcare Hamilton, Hamilton, Ontario, Canada; k School of Mechatronic Systems Engineering, Simon Fraser University, Vancouver, British Columbia, Canada; l Neurobiology Research Unit, University Hospital Rigshospitalet, Copenhagen, Denmark; m Department of Clinical Medicine, Faculty of Health and Medical Sciences, University of Copenhagen, Copenhagen, Denmark; n Department of Psychiatry, Psychiatric Centre Copenhagen, Copenhagen, Denmark; o Department of Computer Science, University of Copenhagen, Copenhagen, Denmark; p Department of Psychiatry, University of Oxford, Oxford, United Kingdom; q Oxford Health NHS Foundation Trust, Warneford Hospital, Oxford, United Kingdom; r Mathison Centre for Mental Health Research and Education, University of Calgary, Calgary, Alberta, Canada; s Department of Psychiatry, Cumming School of Medicine, University of Calgary, Calgary, Alberta, Canada; t Department of Psychiatry, University Health Network, Toronto, Ontario, Canada; u Division of Psychiatry, Centre for Clinical Brain Sciences, Royal Infirmary Edinburgh, Edinburgh, United Kingdom; v Department of Radiology, Taihe Hospital, Hubei University of Medicine, Shiyan, China; w Centre for Depression and Suicide Studies, Unity Health Toronto, Toronto, Ontario, Canada; x Meditation Research Program, Department of Psychiatry, Massachusetts General Hospital, Harvard Medical School, Boston, Massachusetts; y Laureate Institute for Brain Research, Tulsa, Oklahoma; z Penn Statistics in Imaging and Visualization Endeavor Center, Department of Biostatistics, Epidemiology and Informatics, University of Pennsylvania, Philadelphia, Pennsylvania; aa Department of Psychiatry, University of California San Francisco, San Francisco, California; ab Department of Medical Biophysics, University of Toronto, Toronto, Ontario, Canada; ac Department of Radiology and Biomedical Imaging, University of California San Francisco, San Francisco, California; ad School of Psychology, Southwest University, Chongqing, China; ae Department of Neuroimaging, Institute of Psychiatry, Psychology and Neuroscience, King’s College London, London, United Kingdom; af Division of Neuroscience and Experimental Psychology, University of Manchester, Manchester, United Kingdom; ag Department of Psychiatry and Behavioral Sciences, Emory University School of Medicine, Atlanta, Georgia; ah Department of Psychology, Emory University, Atlanta, Georgia; ai Department of Psychology, Stanford University, Stanford, California; aj Generation Scotland, Centre for Medical Informatics, University of Edinburgh, Edinburgh, United Kingdom; ak Department of Psychological and Cognitive Sciences, Tsinghua University, Beijing, China; al Department of Psychiatry, Imperial College London, London, United Kingdom; mm National Institute for Health and Care Research, Maudsley Biomedical Research Centre, London, United Kingdom

**Keywords:** Antidepressant treatment response, Electrophysiological predictors, Major depressive disorder, Multimodal neuroimaging, Neuroanatomical dimensions, Resting-state EEG

## Abstract

**Background:**

Major depressive disorder (MDD) is heterogeneous in clinical presentation and treatment response. The COORDINATE-MDD consortium identified two magnetic resonance imaging (MRI)–derived neuroanatomical profiles: dimension 1 (D1), with relatively preserved gray and white matter, and dimension 2 (D2), showing widespread reductions aligned with immunometabolic profile. Profiles were associated with distinct responses to selective serotonin reuptake inhibitor (SSRI) antidepressant and placebo (PLA). In this study, we examined electrophysiological correlates of the neuroanatomical profiles and their relationship to treatment outcome.

**Methods:**

Baseline resting-state, eyes-closed electroencephalography (EEG) was acquired from 237 medication-free participants with MDD who were in a current depressive episode (155 women; mean age [SD] = 37.47 [13.36] years) from CAN-BIND (Canadian Biomarker Integration Network in Depression) (SSRI) and EMBARC (Establishing Moderators and Biosignatures of Antidepressant Response in Clinical Care) (SSRI or PLA). EEG features included spectral power, frontal alpha asymmetry (FAA), multiscale sample entropy, and intersite phase clustering. Effects of profile (D1 and D2) and clinical outcome (responder, nonresponder; defined as ≥50% symptom improvement) were examined with age, sex, and site as covariates.

**Results:**

No significant electrophysiological differences were observed after covariate adjustment. However, among participants who subsequently responded to treatment, D1 showed greater baseline alpha power in frontal and central regions and lower relative delta posteriorly compared with D2. In PLA-treated responders, D2 showed spectral slowing, elevated low-frequency power, reduced gamma, and coarse-scale entropy compared with D1. Baseline FAA was lower in responders than nonresponders, independent of the neuroanatomical profile.

**Conclusions:**

EEG differences between MRI-defined neuroanatomical profiles emerged in relation to clinical outcome. D1 was associated with electrophysiological patterns consistent with flexible, globally regulated cortical dynamics in SSRI responders, whereas D2 showed a distinct pattern in PLA responders, indicating partially separable neural mechanisms underlying pharmacological and PLA treatment effects.

## Plain Language Summary

Depression is a common condition, but people differ in their symptoms, underlying biology, and response to treatment. This makes it difficult to predict which treatments will be most effective for each individual. Previous research from the COORDINATE-MDD consortium identified two brain-based groups of individuals with depression using MRI scans. One group showed relatively preserved brain structure and better response to antidepressant medication, whereas the other showed more widespread structural changes and similar improvement with medication or placebo, suggesting different underlying mechanisms. In this study, we examined whether these groups also differ in brain activity measured using electroencephalography before treatment. No clear differences were seen across all participants. However, among those who later improved, distinct patterns emerged: one group showed more organized and adaptable brain activity patterns and greater response to medication, whereas the other showed slower activity and reduced complexity linked to placebo response. These findings suggest that different biological mechanisms underlie treatment response, supporting more personalized approaches to care.

Major depressive disorder (MDD) is a leading contributor to the global burden of disease (1) and is clinically highly heterogeneous, with substantial variation in symptoms, illness trajectories, and treatment response (2,3). Because current diagnostic frameworks are symptom based rather than mechanism based, MDD remains a syndrome with poorly defined pathophysiologies. Currently, no biomarker reliably predicts treatment response at the individual level (4).

To address this, we have been investigating the neurobiological heterogeneity of MDD through data-driven machine learning approaches within the COORDINATE-MDD consortium (4). This international multisite initiative integrates harmonized neuroimaging and deep clinical phenotyping to enable biologically informed stratification. The consortium was established to construct a dimensional neuroanatomical-neurofunctional coordinate system for MDD through integrated analysis of raw individual-level neuroimaging in medication-free participants in a current depressive episode of first-episode (FE) or recurrent MDD, combined with deep clinical phenotyping and prospective longitudinal treatment studies to enable individual-level biomarker-based stratification (4). Using the semisupervised HYDRA algorithm, we identified 2 neuroanatomical profiles (dimension 1 [D1] and dimension 2 [D2]) in medication-free participants with MDD (5). D1 was characterized by relatively preserved gray and white matter volumes and showed greater response to selective serotonin reuptake inhibitor (SSRI) antidepressant treatment compared with placebo (PLA), whereas D2 was characterized by widespread subtle volumetric reductions and showed a limited response to either SSRI or PLA (5).

Their robustness was supported by external validation in 37,235 UK Biobank participants, demonstrating generalizability across clinical and general population-based samples (6). D2 was associated with a more adverse biological and clinical profile, including increased systemic inflammation and metabolic dysregulation and increased rates of self-harm and greater exposure to early-life adversity (6). D2 showed convergence with an immunometabolic subtype of depression characterized by elevated inflammation, metabolic dysregulation, and greater illness chronicity, identified in the NESDA (Netherlands Study of Depression and Anxiety) (7). The immunometabolic subtype aligned with the biological and clinical profile captured by D2 in our magnetic resonance imaging (MRI)–defined framework (5,6).

Resting-state electroencephalography (EEG) provides a complementary measure of brain function by capturing large-scale cortical dynamics with millisecond temporal resolution. Baseline EEG features can predict antidepressant response. An EEG-based antidepressant treatment-response classifier in MDD, developed and validated in the large deeply phenotyped cohorts from the CAN-BIND (Canadian Biomarker Integration Network in Depression) (8,9) and EMBARC (Establishing Moderators and Biosignatures of Antidepressant Response in Clinical Care) studies (10), included baseline spectral and complexity features (11). The model achieved a balanced accuracy of 64.2% in internal validation in CAN-BIND and 63.7% in the EMBARC SSRI treatment arm but dropped to chance accuracy in the EMBARC PLA arm (48.7%), demonstrating specificity for pharmacological treatment, with accuracy falling to chance for PLA treatment (11). This suggests that EEG-based predictors may reflect underlying biological variability that is not explicitly modeled.

Informed by this prior work, we focused on 4 classes of resting-state EEG features with established relevance to MDD and antidepressant treatment response. First, band-limited spectral power indices across delta to gamma frequencies capture oscillatory dynamics widely implicated in MDD and treatment outcome (12,13). Spectral power indices predicted escitalopram response in CAN-BIND (14) and formed part of the CAN-BIND/EMBARC SSRI-response classifier (11). Second, frontal alpha asymmetry (FAA) assesses hemispheric differences in frontal cortical activity relevant to affective and motivational processing. Meta-analytic evidence suggests modest and somewhat inconsistent FAA differences in depression (15), whereas alpha/asymmetry features have been linked to antidepressant response and cross-cohort SSRI-response prediction (11,16). Third, multiscale entropy (MSE) quantifies neural signal complexity across time scales, indexing the adaptability of neural dynamics (17). MSE features have been associated with antidepressant and brain-stimulation treatment outcomes and were also included in the CAN-BIND/EMBARC SSRI-response model (11). Finally, intersite phase clustering (ISPC) captures phase-based functional connectivity, reflecting large-scale network integration (18,19). In EMBARC, pretreatment EEG signatures predicted sertraline-specific improvement relative to PLA (20).

In the current study, we extended our prior MRI-based work by examining whether these neuroanatomical profiles (D1/D2) are reflected in baseline resting-state EEG in the same participants from COORDINATE-MDD (4, 5, 6). The D1/D2 assignments were derived in the prior MRI analysis of the same cohort (5) and were not reestimated here. We sought to examine whether the structural MRI-defined profiles exhibit distinct electrophysiological patterns, providing a functional correlate of structural heterogeneity. Eyes-closed, resting-state EEG recordings were obtained from the multisite CAN-BIND (8,9) and EMBARC (10) clinical trials within the COORDINATE-MDD consortium. The same 2 cohorts contributed the EEG data analyzed here. We note this overlap explicitly, while the current analysis has a distinct aim to use EEG features to interrogate MRI-defined neuroanatomical dimensions established in an independent semisupervised machine learning (HYDRA) analysis in the same participants (5). We sought to examine the baseline EEG markers of treatment response and whether these predictive effects differ between pharmacological (SSRI) and PLA treatments. By analyzing the total sample and then stratifying by treatment, we aimed to determine whether EEG markers reflect a common signal of response or distinct neural signatures specific to each treatment type.

## Methods and Materials

### Datasets

Data were integrated from 2 large multisite clinical trials, CAN-BIND (8,9) and EMBARC (10), within the COORDINATE-MDD consortium (4). Ethical approval was obtained by the respective research ethics boards, and all participants provided written informed consent. The current EEG analysis included the same participants previously characterized in the COORDINATE-MDD structural MRI study (4,5). In that study, individual patients were assigned to 2 MRI-defined neuroanatomical profiles using the semisupervised HYDRA clustering algorithm, which separates patients from healthy control participants while identifying subgroups within the patient cohort. This approach yields categorical assignments (D1 and D2) rather than continuous factor scores. D1 was characterized by relatively preserved gray and white matter volumes and showed greater clinical improvement following SSRI treatment, whereas D2 was characterized by widespread subtle reductions in gray and white matter and limited response to SSRI or PLA. These HYDRA-derived dimension labels were directly carried forward into the current EEG analysis without reestimation.

The initial dataset comprised 250 participants with MDD (CAN-BIND: 54; EMBARC: 196) with both neuroanatomical dimension classification (D1, D2) and EEG data. After excluding 13 participants for data quality issues, the final sample comprised 237 individuals: D1: 116 participants with MDD (70 women; mean [SD] age = 37.58 [13.25] years) and D2: 121 participants with MDD (85 women; mean [SD] age 37.36 [13.51] years). Within CAN-BIND, there were 15 participants with MDD (9 women) in D1 and 39 participants with MDD (29 women) in D2. Within EMBARC, there were 101 participants with MDD (61 women) in D1 and 82 participants with MDD (56 women) in D2 (Table 1).

**Table 1:** Demographic Characteristics of Participants

Demographic Feature	CAN-BIND		EMBARC	
	Dimension 1, *n* = 15	Dimension 2, *n* = 39	Dimension 1, *n* = 101	Dimension 2, *n* = 82
Women	9	29	61	56
Age, Years	34.20 (14.11)	38.72 (12.96)	38.08 (13.12)	36.72 (13.8)
Education, Years	13.33 (1.54)	13.79 (2.61)	15.5 (2.49)	14.98 (2.55)
Race				
White	11	27	84	74
Non-White	4	12	24	14
First-Episode MDD	7	14	0	1
Recurrent MDD	8	24	108	87
Depressive Severity, Baseline				
MADRS	29.08 (5.66)	30.39 (5.28)	NA	NA
HAMD-17	NA	NA	19.42 (3.82)	19.68 (3.63)
Treatment				
Antidepressant medication	15	39	54	33
Placebo	NA	NA	47	49

Values represent *n* or mean (SD). Depression severity was assessed using the MADRS in CAN-BIND and the HAMD-17 in EMBARC. In CAN-BIND, all participants received escitalopram. In EMBARC, participants were randomized to receive either sertraline or placebo.

CAN-BIND, Canadian Biomarker Integration Network in Depression; EMBARC, Establishing Moderators and Biosignatures of Antidepressant Response in Clinical Care; HAMD-17, 17-item Hamilton Depression Rating Scale; MADRS, Montgomery–Åsberg Depression Rating Scale; MDD, major depressive disorder; NA, not applicable.

CAN-BIND and EMBARC differed substantially in demographic composition. In CAN-BIND, 38 of 54 participants (70.4%) identified as White and 16 (29.6%) as non-White, whereas in EMBARC, 158 of 197 participants (80.2%) identified as White and 39 (19.8%) as non-White. This distribution differed significantly across cohorts (χ^2^_1_ = 4.03, *p* = .045). Episode recurrence showed an even stronger cohort dependence; in CAN-BIND, 21 participants (39.6%) were experiencing an FE and 32 (60.4%) a recurrent episode, whereas EMBARC included 1 FE participant (0.5%) and 183 recurrent cases (99.5%). This difference was highly significant (χ^2^_1_ = 73.21, *p* < .0001). In contrast, neither age (CAN-BIND: mean [SD] age 37.41 [13.76] years; EMBARC: mean [SD] age 37.45 [13.35] years; *t*_235_ = 0.02, *p* = .986) nor sex distribution (CAN-BIND: 38.9% female; EMBARC: 59.4% female; χ^2^_1_ = 2.68, *p* = .102) differed between cohorts. These patterns indicate that race and recurrence are not independent participant-level confounders but are structurally embedded in cohort/site membership, arising from study-specific recruitment strategies rather than the neuroanatomical dimensions under study.

In CAN-BIND, participants received open-label escitalopram (10–20 mg/day) for 8 weeks. In EMBARC, participants were randomized to sertraline (50–200 mg/day; D1: 54; D2: 33) or PLA (D1: 47; D2: 49) for 8 weeks. Treatment response was achieved in D1 for 50 participants (34 women) and in D2 for 48 participants (34 women); persistent symptoms were present in D1 in 66 participants (36 women) and in D2 in 73 participants (51 women) (Table 2). The complete protocol and clinical details are available in the Supplemental Methods.

**Table 2:** Demographic Characteristics by Dimension (D1, D2) and Treatment Response Status

	Dimension 1		Dimension 2	
	Treatment Response, *n* = 50	Persistent Symptoms, *n* = 66	Treatment Response, *n* = 48	Persistent Symptoms, *n* = 73
Women	34	36	34	51
Age, Years	35.02 (12.04)	39.52 (13.87)	37.42 (14.42)	37.33 (12.98)
Year of Education	15.32 (2.32)	15.14 (2.63)	14.43 (2.81)	14.71 (2.51)
HAMD Baseline	19.74 (4.00)	19.40 (3.64)	20.71 (3.79)	20.47 (4.19)
HAMD Posttreatment	4.90 (2.93)	17.05 (4.90)	5.79 (3.62)	16.29 (4.60)
Treatment				
EMBARC placebo	16	31	19	30
EMBARC medication	29	25	14	19
CAN-BIND medication	5	10	15	24

Values represent *n* or mean (SD). Depression severity was assessed using the MADRS in CAN-BIND and the HAMD-17 in EMBARC. HAMD-17 scores for CAN-BIND were estimated from MADRS scores using equipercentile linking (37) for harmonization. Original MADRS baseline and posttreatment values for CAN-BIND are in Table 1. In CAN-BIND, participants received escitalopram. In EMBARC, participants were randomized to sertraline or placebo.

CAN-BIND, Canadian Biomarker Integration Network in Depression; EMBARC, Establishing Moderators and Biosignatures of Antidepressant Response in Clinical Care; HAMD-17, 17-item Hamilton Depression Rating Scale; MADRS, Montgomery–Åsberg Depression Rating Scale.

### EEG Data Recording

Resting-state EEG was acquired at baseline prior to treatment initiation. In CAN-BIND, participants completed an 8-minute eyes-closed resting-state EEG recording across 4 sites (8,9). In EMBARC, EEG was recorded in four 2-minute blocks alternating between eyes-open and eyes-closed conditions (10); only eyes-closed segments were included for consistency with CAN-BIND.

### EEG Data Preprocessing

EEG data were segmented into 2-second epochs and filtered using standard procedures. Artifact-contaminated epochs and channels were identified using amplitude-based criteria, and participants with excessive data loss (fewer than 20 usable channels or >50% channel rejection) were excluded. To ensure data quality, a voltage deflection threshold approach was applied for identifying and rejecting noisy epochs. A moving 80-ms window was used to compute the difference between the maximum and minimum voltage within each epoch. Epochs were flagged as potentially noisy if any channel exhibited a voltage deflection exceeding 100 μV. Channels were identified as artifactual and removed if they exceeded this 100 μV threshold in more than 50% of epochs. Following channel removal, epochs were excluded if more than 25% of remaining channels exceeded the same threshold.

Participants were excluded from further analysis if they retained fewer than 20 usable channels after preprocessing or if more than 50% of channels were rejected. These criteria resulted in the exclusion of 13 participants. All recordings were re-referenced to the average reference and interpolated to a standardized 32-channel montage to harmonize data across cohorts (Supplemental Methods).

### Reliability and Data Sufficiency Checks

The reliability of all EEG-derived features was assessed prior to group-level analysis using split-half consistency and intraclass correlation coefficients (ICCs) within each cohort. Each participant’s clean epochs were partitioned into 2 halves by interleaved (odd vs. even) assignment; features were computed independently on each half, and Pearson correlations between halves were calculated across participants for every electrode-by-frequency (or electrode-by-scale) bin and Spearman-Brown corrected to full-length reliability. ICCs were computed using a 2-way mixed-effects, single-measurement, absolute-agreement model (ICC_3,1_) on the same 2 halves, interpreted using conventional ranges. Mean ICCs were excellent for absolute spectral power (mean 0.96, range 0.94–0.997), high for log-transformed power and FAA (≥0.85), good for MSE (mean ≈ 0.78), and acceptable for ISPC (mean ≈ 0.72) (Table S5). Features with mean ICC < 0.50 were flagged but retained.

### EEG Feature Extraction

Resting-state EEG features included spectral power, FAA, MSE, and ISPC. Features were computed using MATLAB (version R2024b; The MathWorks, Inc.) scripts adapted from the Sheffield Autism Biomarkers toolbox, previously validated in large-scale clinical EEG studies. Spectral power was estimated across canonical frequency bands (delta, theta, alpha, beta, and gamma). FAA indexed hemispheric frontal asymmetry in alpha-band activity. MSE quantified signal complexity across multiple temporal scales, and ISPC assessed phase-based functional connectivity between electrode pairs. Regional EEG features were derived by averaging channelwise measures within predefined anatomical groupings and by computing hemispheric asymmetry indices. Full computational details and parameter settings are provided in the Supplemental Methods.

### Statistical Analysis of EEG-Derived Features

EEG-derived features were analyzed using 2 complementary approaches: 1) feature-level analyses of covariance (ANCOVAs) on predefined regional measures and 2) electrodewise threshold-free cluster enhancement (TFCE) to characterize spatially distributed effects across channels and frequencies or time scales. To minimize potential confounding arising from demographic or study-related differences, statistical models included age, sex, and study site as covariates. These covariates were selected based on empirical examination of the sample; demographic characteristics such as race/ethnicity and episode recurrence were strongly patterned by cohort membership rather than varying independently across individuals, and therefore adjusting for them in addition to site would have introduced redundancy and potential multicollinearity. Thus, site served as the appropriate cohort-level adjustment after EEG harmonization.

### Feature-Level Analyses (ANCOVA)

Feature-level comparisons were conducted using 2-way ANCOVA, with neuroanatomical dimension (D1 and D2) and treatment outcome status (responder, defined as ≥50% improvement in depressive symptoms from baseline, and nonresponder, defined as having persistent symptoms with <50% improvement from baseline) as fixed factors and age, sex, and site as covariates.

Feature-level analyses were conducted to examine regional electrophysiological differences between neuroanatomical dimensions. For each of the 335 regionally averaged EEG features (spectral power, asymmetry, MSE, and IPSC), we fit an ANCOVA model of the form: EEG feature ∼ dimension × responder + age + sex + site.

Age, sex, and study site (CAN-BIND vs. EMBARC) were included as covariates to adjust for demographic and cohort-level influences on EEG signals after harmonization. All features were *z*-scored prior to analysis. Effect sizes were quantified using partial eta-squared (η_p_^2^), and *p* values for the dimension term were corrected using the Benjamini-Hochberg false discovery rate (FDR) across all features.

Treatment type (SSRI vs. PLA) was not included in the primary ANCOVA because treatment allocation was cohort specific, creating structural dependence between treatment and site. Instead, treatment-related effects were examined in subgroup analyses conducted separately within antidepressant medication (ADM) and PLA arms using the same model structure. Sensitivity analyses using a reduced covariate set (age + site) produced highly consistent results, indicating that the inclusion of sex did not materially influence inference. Full ANCOVA outputs, effect-size distributions, and FDR-adjusted results are provided in the Supplement. All analyses were performed in MATLAB.

Given the strong dependence of episode recurrence on study cohort, recurrence was not included as a covariate in primary models. Instead, episode-stratified sensitivity analyses were conducted in FE and recurrent samples using the same model structure.

### Electrodewise Analyses: TFCE

Electrodewise analyses were conducted using TFCE, a nonparametric cluster-based approach that accounts for spatial and spectral dependencies without requiring arbitrary cluster-forming thresholds, thereby providing sensitivity to weak but spatially or temporally extended effects (21,22). Prior to TFCE estimation, electrode-frequency (or electrode-scale) matrices were residualized for age, sex, and study site at each bin to ensure that contrasts reflected neuroanatomical dimension and clinical outcome rather than residual demographic or cohort-related variance following harmonized preprocessing and standardized 32-channel montage interpolation.

Permutation inference was implemented using the ept_TFCE MATLAB toolbox (https://github.com/Mensen/ept_TFCE-matlab) with 5000 2-tailed Monte Carlo permutations and recommended TFCE parameters (height exponent H = 2; extent exponent E = 0.66) (22). TFCE was applied separately to spectral power, MSE, and ISPC, yielding cluster-enhanced statistical maps and permutation-derived *p* values for each electrode-frequency or electrode-scale bin. All contrasts mirrored those in the feature-level analyses, including D1 versus D2, responder versus nonresponder, and the 4 dimension-by-outcome subgroup comparisons. These contrasts were evaluated in the total sample and separately within the ADM and PLA subgroups to permit treatment-specific interpretation without introducing collinearity arising from cohort-dependent treatment allocation. Significant TFCE effects were visualized using channel-by-frequency heatmaps and scalp topographies summarizing the spatial distribution of significant bins.

As a sensitivity analysis, TFCE was also repeated within FE-only and recurrent-episode (recurrent-only) subgroups using the same residualization strategy (age, sex, and site) and contrast set. These episode-stratified analyses were conducted to assess whether dimension-by-responder effects observed in the total sample were driven by episode status.

## Results

### Dimension Effects in the Total Sample

Across the entire cohort, 68 EEG features showed nominal dimension effects at an uncorrected threshold (*p* < .05), but none survived FDR correction across the 335 features analyzed. After adjusting for age, sex, and site, there were no significant global differences between D1 and D2 in absolute or relative spectral power, MSE, or ISPC. Complete ANCOVA results are presented in Table S1. Covariate-adjusted TFCE analyses likewise yielded no channel- or frequencywise clusters differentiating the dimensions (Table S3). FAA did not differ between D1 and D2 (*F*_1,227_ = 0.25, *p* = .620, η_p_^2^ = 0.001) (Table S1), further indicating minimal baseline electrophysiological divergence at the total sample level.

### Responder Status and Outcome-Dependent Dimension Effects

Responder status was not associated with any FDR-corrected feature-level differences. FAA was the only univariate measure showing an association with clinical outcome, with responders exhibiting lower FAA than nonresponders (*F*_1,227_ = 5.10, *p* = .025, η_p_^2^ = 0.022) (Table S1).

**Figure 1:**
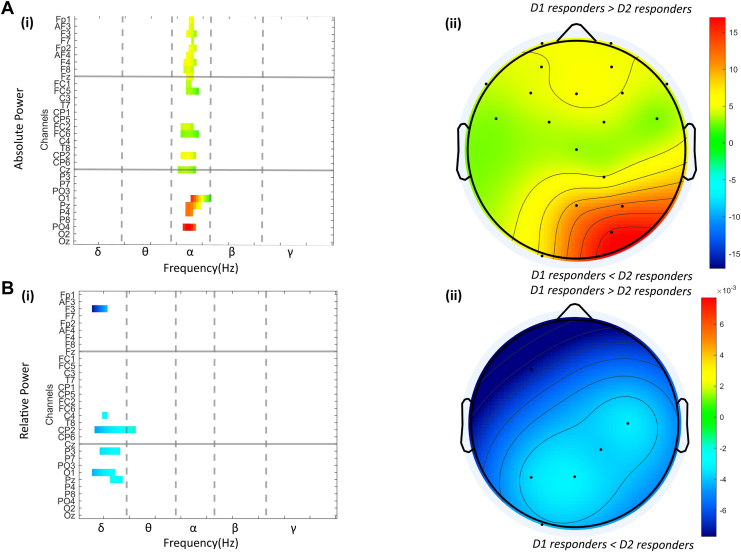
Resting-state electroencephalography (EEG) power differences between neuroanatomical dimensions among treatment responders. Panels **(Ai)** and **(Bi)** show frequency-by-channel representations of statistically significant group differences between dimension 1 (D1) responders and dimension 2 (D2) responders in absolute power **(A)** and relative power **(B)**, respectively, across canonical frequency bands (δ, θ, α, β, and γ). Significant effects were identified using threshold-free cluster enhancement (TFCE) (5000 permutations), controlling for age, sex, and study site. Panels **(Aii)** and **(Bii)** display corresponding scalp topographies illustrating the channelwise mean differences between D1 and D2 responders, averaged across frequency bins that contributed to significant TFCE effects. Warmer colors indicate greater power in D1 responders compared with D2 responders, whereas cooler colors indicate greater power in D2 responders. D1 responders exhibited higher absolute alpha power across frontal, central, and parieto-occipital regions alongside lower relative delta power over posterior regions compared with D2 responders. No significant differences were observed between dimensions among nonresponders.

A TFCE effect emerged in the contrast between D1 and D2 among responders in the total sample. After adjustment for age, sex, and site, D1 responders exhibited greater absolute alpha power across frontal and central clusters and lower relative delta power in posterior clusters relative to D2 responders (TFCE-corrected *p* < .05) (Figure 1; Table S3).

No differences were observed between D1 and D2 among nonresponders.

### Dimension Differences Emerging in the PLA Subgroup

Although the pooled sample showed no dimension-level differences, a coherent pattern emerged when analyses were restricted to the PLA subgroup. Detailed ANCOVA results are provided in Table S2. D2 consistently exhibited higher relative low-frequency power, particularly theta, alpha, and delta across centroparietal, occipitoparietal, frontal, and central regions, whereas D1 showed higher low-gamma activity and greater MSE.

Specifically, D2 showed markedly higher centroparietal alpha power (D1 mean = −0.284; D2 mean = 0.242; *t*_90_ = −3.52, *p* = 6.7 × 10^-4^, *q* = .0022), higher frontal alpha (D1 mean = −0.311; D2 mean = 0.209; *t*_90_ = −3.17, *p* = .0021, *q* = .0032), and higher occipitoparietal theta (D1 mean = −0.260; D2 mean = 0.252; *t*_90_ = −3.22, *p* = .0018, *q* = .0032). Central theta followed the same pattern (D1 mean = −0.262; D2 mean = 0.220; *t*_90_ = −3.43, *p* = 9.1 × 10^-4^, *q* = .0027). Conversely, D1 demonstrated greater gamma-band activity, particularly in right centroparietal regions (D1 mean = 0.275; D2 mean = −0.239; *t*_90_ = 3.24, *p* = .0017, *q* = .0032), and higher coarse-scale entropy in the bilateral frontal cortex (e.g., left frontal coarse MSE: D1 mean = 0.333; D2 mean = −0.176; *t*_90_ = 3.68, *p* = 3.9 × 10^-4^, *q* = .0022).

The dimension differences observed in the PLA subgroup were not replicated in TFCE analyses (Table S1). This is expected because ANCOVA aggregates signals across regions, increasing sensitivity to broad effects, whereas TFCE tests thousands of pointwise comparisons and detects only spatially contiguous clusters. The PLA effects were consistent in direction but spatially diffuse, making them detectable at the regional level but not sufficiently focal for TFCE significance.

### ADM Subgroup

In the antidepressant-treated group, neither feature-level analyses nor TFCE identified covariate-adjusted dimension or responder differences (Tables S1 and S3).

### Comparison With Unadjusted Analyses

Unadjusted TFCE analyses replicated several previously reported effects, including widespread D1 elevations in alpha and beta power and higher coarse-scale MSE, but none remained significant following adjustment for age, sex, and site (Table S3). The contrast between unadjusted and covariate-adjusted results underscores the substantial contribution of demographic and cohort-level variance to apparent EEG differences in multisite datasets.

### Sensitivity Analyses

Including sex as an explicit covariate in all ANCOVA and TFCE models did not alter the direction or significance of any findings; all dimension-, responder-, and treatment-related results remained unchanged after accounting for sex (Table S2).

We examined whether the main dimension-by-responder findings were driven by episode status, repeating the ANCOVA and TFCE analyses within FE-only and recurrent-only subgroups using the same main model structure (dimension × responder + age + sex + site) and contrast set. In both episode-restricted samples, no effects survived FDR correction at the feature level or TFCE correction at the electrodewise level. Importantly, the directions of the estimated effects in these subsets were consistent with the full-sample pattern (i.e., higher alpha power and lower relative delta power in D1 responders compared with D2 responders), but confidence intervals were wide and clusterwise TFCE statistics did not reach significance, consistent with limited power in the smaller FE-only and recurrent-only samples. These results suggest that the main dimension-by-responder findings are not solely driven by episode status but that episode-stratified analyses are underpowered to detect these effects after correction (Table S2).

## Discussion

In the current study, we examined whether the MRI-derived neuroanatomical dimensions established in COORDINATE-MDD are reflected in baseline electrophysiological activity. Across the full cohort, differences between D1 and D2 were minimal once age, sex, and site were controlled. A different pattern emerged when analyses focused on individuals who subsequently demonstrated clinical improvement. In the responder subgroup, D1 showed significantly greater absolute alpha power and lower relative delta power across frontal, central, and parieto-occipital regions than D2. A complementary pattern was evident in the PLA arm, in which D2 expressed elevated low-frequency power (theta, alpha, delta) and reduced gamma activity and coarse-scale MSE at baseline compared with D1. Importantly, these differences were preserved with and without covariate adjustment and did not appear among nonresponders, suggesting that the neural expression of MRI-derived dimensions is electrophysiologically detectable when the analysis is conditioned on treatment context in individuals who subsequently respond to active treatment and among those receiving PLA.

Increases in alpha activity are associated with top-down inhibitory control that facilitates selective attention by suppressing task-irrelevant brain areas (23). Alpha oscillations are typically associated with global corticocortical dynamics, whereas delta oscillations index more locally dominant neocortical processes (24,25). The combination of higher baseline alpha and lower relative delta in D1 responders is consistent with a shift toward more globally regulated and flexible cortical states. Increased baseline alpha power has predicted treatment response across pharmacological and neuromodulatory interventions, including SSRI (16) and repetitive transcranial magnetic stimulation (26), and an alpha-based EEG signature selectively predicted response to the SSRI sertraline but not PLA in EMBARC (19). Reduced baseline low-frequency power has similarly been reported as a predictor of antidepressant and psychotherapy response, including relative theta for SSRI/serotonin-norepinephrine reuptake inhibitor (SNRI) treatment (27) and relative delta for cognitive behavioral therapy (CBT) (28). The current finding extends this literature by linking the alpha-rich, low-delta profile within the structurally preserved D1, providing a multimodal anchor for what had been described as a spatially distributed electrophysiological marker of treatment response.

FAA showed a main effect of clinical response status, with responders exhibiting lower FAA at baseline, reflecting greater left-lateralized frontal alpha activity. This pattern aligns with the approach-withdrawal model of affective motivation (29) and converges with findings from the NeuroPharm trial, in which lower FAA was associated with greater symptom improvement following SSRI treatment (30). Although meta-analytic evidence suggests that FAA effects in MDD are small and heterogeneous (15), the current findings indicate that FAA may index prefrontal regulatory capacity relevant to treatment responsiveness when examined alongside neurobiological stratification. No covariate-adjusted differences were observed in MSE or ISPC, either across dimensions or in responder subgroups. These null effects indicate that entropy- and connectivity-based contrasts may be weaker or more variable than previously assumed once demographic factors are rigorously accounted for.

Crucially, these differences were not present among nonresponders, implying that the electrophysiological signature of D1 may not be a universal feature of the dimension but rather one that emerges in individuals whose underlying neurobiology can support clinical improvement. This aligns with the multimodal profile of D1, characterized by relatively preserved cortical structure, lower systemic inflammation, and fewer immunometabolic disturbances (6). By contrast, D2, marked by subtle but widespread cortical reductions, increased systemic inflammation, metabolic disturbances, increased rates of self-harm, and a history of greater childhood adversity, may reflect a less flexible and more constrained neurophysiological phenotype for which spectral modulation is less predictive of symptom change (5,6).

Further insight arises from examining the PLA subgroup. In prior work, D2 showed comparable clinical improvement following SSRI and PLA treatment with limited separation between active medication and PLA, suggesting a greater contribution of nonspecific or expectancy-related mechanisms (5). In the current EEG subsample, D2 in the PLA arm showed pronounced spectral slowing, characterized by elevated low-frequency power, reduced gamma activity, and lower MSE. This constellation of features aligns with markers of reduced neural flexibility and metabolic or inflammatory burden, in which increased low-frequency power, reduced high-frequency activity, and reduced complexity track accelerated biological brain aging (31, 32, 33, 34). In contrast, D1 showed greater gamma activity and higher frontal entropy, consistent with more dynamically reconfigurable neural networks. These PLA-restricted findings suggest that neural dynamics in D2 may be relatively insensitive to pharmacological modulation, whereas D1 may exhibit treatment-dependent modulation that becomes evident only among responders.

The present findings complement prior work showing that baseline EEG features can predict antidepressant response in CAN-BIND and EMBARC (11), while extending this literature by situating treatment-relevant electrophysiological signals within MRI-defined neurobiological heterogeneity. Features previously associated with antidepressant response, including higher alpha power and reduced low-frequency activity, were predominantly expressed in D1, a structurally more preserved dimension characterized by greater SSRI responsiveness. In contrast, D2 showed spectral slowing and reduced signal complexity among PLA responders, suggesting that nonspecific clinical improvement may be supported by distinct neural dynamics. This interpretation is consistent with EMBARC evidence that resting-state EEG measures relate to separable depressive endophenotypes, including neuroticism, reward learning, and cognitive-control impairment (35). Together, these findings suggest that EEG predictors of treatment response are unlikely to reflect a single uniform biomarker but may instead represent dimension-specific functional expressions of underlying neurobiological heterogeneity.

Limitations include the sample size constrained by available resting-state EEG and the use of resting-state rather than task-based recording, such as during emotional or cognitive tasks, which might provide additional dynamic EEG differences relevant to dimensions and treatment responsiveness (36). Although EEG-derived features such as spectral power and entropy are scalable in clinical settings, their translational utility requires prospective validation, normative reference ranges, and harmonized multisite protocols. Integrating EEG with structural and clinical features may ultimately support stratified or precision treatment approaches. Although sex was included as a covariate, sex-specific effects on neuroanatomical or electrophysiological profiles were not examined directly. Future studies with larger samples may assess potential interactions between sex, neurobiological dimensions, and treatment response. EEG preprocessing was harmonized across cohorts, although acquisition hardware and recording environments might have introduced residual variance that could not be fully accounted for. However, cohort-adjusted sensitivity analyses showed consistent effects. Finally, the predictive dimension-by-responder effects should be validated prospectively to assess their applicability in stratified treatment approaches.

### Conclusions

Baseline electrophysiological differences between HYDRA-defined neuroanatomical dimensions are not evident across the full MDD sample after accounting for demographic and cohort effects. However, when examined in relation to subsequent outcome, distinct patterns emerged. D1 was associated with a baseline EEG profile of higher alpha and lower relative delta power in individuals who showed a response to SSRI treatment. In contrast, within the PLA arm, D2 showed a different electrophysiological pattern, spectral slowing, reduced gamma activity, and lower signal complexity, indicating that PLA response is supported by distinct neural dynamics rather than representing a weaker form of pharmacological response. These findings suggest that EEG markers of treatment response reflect the functional expression of underlying neurobiological heterogeneity rather than a single uniform biomarker and that pharmacological and PLA-related improvements arise from partially separable mechanisms. This supports the development of mechanism-informed, stratified approaches to treatment selection in MDD.

## Supplementary Material

Tables S1–S5

Key Resources Table

## References

[1] Yan G., Zhang Y., Wang S., Yan Y., Liu M., Tian M., Tian W. (2024). Global, regional, and national temporal trend in burden of major depressive disorder from 1990 to 2019: An analysis of the global burden of disease study. Psychiatry Res.

[2] Fried E.I., Nesse R.M. (2015). Depression is not a consistent syndrome: An investigation of unique symptom patterns in the STAR∗D study. J Affect Disord.

[3] Fu C.H.Y., Fan Y., Davatzikos C. (2019). Addressing heterogeneity (and homogeneity) in treatment mechanisms in depression and the potential to develop diagnostic and predictive biomarkers. NeuroImage Clin.

[4] Fu C.H.Y., Erus G., Fan Y., Antoniades M., Arnone D., Arnott S.R. (2023). AI-based dimensional neuroimaging system for characterizing heterogeneity in brain structure and function in major depressive disorder: COORDINATE-MDD consortium design and rationale. BMC Psychiatry.

[5] Fu C.H.Y., Antoniades M., Erus G., Garcia J.A., Fan Y., Arnone D. (2024). Neuroanatomical dimensions in medication-free individuals with major depressive disorder and treatment response to SSRI antidepressant medications or placebo. Nat Mental Health.

[6] Xiao W., Woodham R.D., Cui Y., Wen J., Antoniades M., Srinivasan D. (2025). Neuroanatomical dimensions in major depression linked to cognition, adverse life events, self-harm, metabolomics and genetics. Commun Med.

[7] Lamers F., Milaneschi Y., Vinkers C.H., Schoevers R.A., Giltay E.J., Penninx B.W.J.H. (2020). Depression profilers and immuno-metabolic dysregulation: Longitudinal results from the NESDA study. Brain Behav Immun.

[8] Farzan F., Atluri S., Frehlich M., Dhami P., Kleffner K., Price R. (2017). Standardization of electroencephalography for multi-site, multi-platform and multi-investigator studies: Insights from the Canadian Biomarker Integration Network in Depression. Sci Rep.

[9] Lam R.W., Milev R., Rotzinger S., Andreazza A.C., Blier P., Brenner C. (2016). Discovering biomarkers for antidepressant response: Protocol from the Canadian Biomarker Integration Network in Depression (CAN-BIND) and clinical characteristics of the first patient cohort. BMC Psychiatry.

[10] Trivedi M.H., McGrath P.J., Fava M., Parsey R.V., Kurian B.T., Phillips M.L. (2016). Establishing moderators and biosignatures of antidepressant response in clinical care (EMBARC): Rationale and design. J Psychiatr Res.

[11] Schwartzmann B., Dhami P., Uher R., Lam R.W., Frey B.N., Milev R. (2023). Developing an electroencephalography-based model for predicting response to antidepressant medication. JAMA Netw Open.

[12] Tsai Y.-C., Li C.-T., Juan C.-H. (2023). A review of critical brain oscillations in depression and the efficacy of transcranial magnetic stimulation treatment. Front Psychiatry.

[13] Watts D., Pulice R.F., Reilly J., Brunoni A.R., Kapczinski F., Passos I.C. (2022). Predicting treatment response using EEG in major depressive disorder: A machine-learning meta-analysis. Transl Psychiatry.

[14] Baskaran A., Farzan F., Milev R., Brenner C.A., Alturi S., Pat McAndrews M.P. (2018). The comparative effectiveness of electroencephalographic indices in predicting response to escitalopram therapy in depression: A pilot study. J Affect Disord.

[15] van der Vinne N., Vollebregt M.A., van Putten M.J.A.M., Arns M. (2017). Frontal alpha asymmetry as a diagnostic marker in depression: Fact or fiction? A meta-analysis. NeuroImage Clin.

[16] Bruder G.E., Sedoruk J.P., Stewart J.W., McGrath P.J., Quitkin F.M., Tenke C.E. (2008). Electroencephalographic alpha measures predict therapeutic response to a selective serotonin reuptake inhibitor antidepressant: Pre- and post-treatment findings. Biol Psychiatry.

[17] Costa M., Goldberger A.L., Peng C.-K. (2005). Multiscale entropy analysis of biological signals. Phys Rev E.

[18] Cohen M.X. (2014).

[19] Lachaux J.P., Rodriguez E., Martinerie J., Varela F.J. (1999). Measuring phase synchrony in brain signals. Hum Brain Mapp.

[20] Wu W., Zhang Y., Jiang J., Lucas M.V., Fonzo G.A., Rolle C.E. (2020). An electroencephalographic signature predicts antidepressant response in major depression. Nat Biotechnol.

[21] Smith S.M., Nichols T.E. (2009). Threshold-free cluster enhancement: Addressing problems of smoothing, threshold dependence and localisation in cluster inference. NeuroImage.

[22] Mensen A., Khatami R. (2013). Advanced EEG analysis using threshold-free cluster-enhancement and non-parametric statistics. NeuroImage.

[23] Jensen O., Mazaheri A. (2010). Shaping functional architecture by oscillatory alpha activity: Gating by inhibition. Front Hum Neurosci.

[24] Nunez P.L. (1995).

[25] Nunez P.L., Srinivasan R. (2006). A theoretical basis for standing and traveling brain waves measured with human EEG with implications for an integrated consciousness. Clin Neurophysiol.

[26] Noda Y., Nakamura M., Saeki T., Inoue M., Iwanari H., Kasai K. (2013). Potentiation of quantitative electroencephalograms following prefrontal repetitive transcranial magnetic stimulation in patients with major depression. Neurosci Res.

[27] Iosifescu D.V., Greenwald S., Devlin P., Mischoulon D., Denninger J.W., Alpert J.E., Fava M. (2009). Frontal EEG predictors of treatment outcome in major depressive disorder. Eur Neuropsychopharmacol.

[28] Schwartzmann B., Quilty L.C., Dhami P., Uher R., Allen T.A., Kloiber S. (2023). Resting-state EEG delta and alpha power predict response to cognitive behavioral therapy in depression: A Canadian Biomarker Integration network in Depression study. Sci Rep.

[29] Davidson R.J., Ekman P., Saron C.D., Senulis J.A., Friesen W.V. (1990). Approach-withdrawal and cerebral asymmetry: Emotional expression and brain physiology: I. J Pers Soc Psychol.

[30] Ip C.-T., Olbrich S., Ganz M., Ozenne B., Köhler-Forsberg K., Dam V.H. (2021). Pretreatment qEEG biomarkers for predicting pharmacological treatment outcome in major depressive disorder: Independent validation from the NeuroPharm study. Eur Neuropsychopharmacol.

[31] Scally B., Burke M.R., Bunce D., Delvenne J.-F. (2018). Resting-state EEG power and connectivity are associated with alpha peak frequency slowing in healthy aging. Neurobiol Aging.

[32] Jeong J. (2004). EEG dynamics in patients with Alzheimer’s disease. Clin Neurophysiol.

[33] Mizuno T., Takahashi T., Cho R.Y., Kikuchi M., Murata T., Takahashi K., Wada Y. (2010). Assessment of EEG dynamical complexity in Alzheimer’s disease using multiscale entropy. Clin Neurophysiol.

[34] Liu H., Zhang Y., Zhang J., Wang Y., Wu Y., Li Y. (2022). QEEG indices are associated with inflammatory and metabolic risk factors in Parkinson’s disease dementia: An observational study. EClinicalmedicine.

[35] Webb C.A., Dillon D.G., Pechtel P., Goer F.K., Murray L., Huys Q.J.M. (2016). Neural correlates of three promising endophenotypes of depression: Evidence from the EMBARC study. Neuropsychopharmacology.

[36] de Freitas S.B., Marques A.A., Bevilaqua M.C., de Carvalho M.R., Ribeiro P., Palmer S. (2016). Electroencephalographic findings in patients with major depressive disorder during cognitive or emotional tasks: A systematic review. Rev Bras Psiquiatr.

[37] Leucht S., Fennema H., Engel R.R., Kaspers-Janssen M., Lepping P., Szegedi A. (2018). Translating the HAM-D into the MADRS and vice versa with equipercentile linking. J Affect Disord.

