# Supplementary material for: Neurophysiological Correlates of Neuroanatomical Dimensions in Major Depressive Disorder: Electroencephalographic Markers of Brain Function and Treatment Outcome
Source: Biol Psychiatry Glob Open Sci. 2026 May 7;6(5):100750. doi: 10.1016/j.bpsgos.2026.100750 (PMC13392939; doi:10.1016/j.bpsgos.2026.100750)
Supplement: Supplemental Methods [file mmc1.pdf]

SUPPLEMENTARY INFORMATION

Neurophysiological Correlates of Neuroanatomical Dimensions in Major Depressive Disorder: Electroencephalographic Markers of Brain Function and Treatment Outcome

Xiao *et al.*

Supplementary Materials

Supplementary Methods

Summary of CAN-BIND and EMBARC prospective clinical trials

CAN-BIND is a multi-site prospective treatment study with six recruitment sites in Canada (1); four of these sites collected the EEG data analysed in the present study. Inclusion criteria included MDD diagnosis, according to DSM-IV-TR criteria, using the Mini-International Neuropsychiatric Interview (MINI), in current depressive episode with Montgomery-Åsberg Depression Rating Scale (MADRS) score of a minimum of 24 and being medication-free. From CAN-BIND, EEG data were available in 138 MDD participants (82 women; mean age 36.24 years, SD 12.80 years) and 54 healthy control participants (35 women; mean age 33.32 years, SD 11.01 years), acquired at baseline before treatment initiation in the MDD participants. All MDD participants received treatment with SSRI antidepressant, escitalopram, for 8 weeks. Treatment response was defined as a ≥50% reduction in MADRS score from baseline to week 8.

In CAN-BIND, participants completed an 8-minute resting-state EEG recording with their eyes closed at baseline (2;3). Recordings were acquired across four sites using EEG systems: Compumedics Neuroscan Synamp at CAMH, Biosemi Active-Two at Toronto General and Western Hospitals (TGH/TWH), QuickAmp system at University of British Columbia (UBC), and EGI NetAmps 300 system with 128-channel HydroCel GSN cap at Queen's University (QNS). Most sites employed a 64-channel setup with an additional four EOG channels using the 10–10 layout, whereas QNS used a 128-channel HydroCel array.

EMBARC is a multi-site prospective, double-blind, randomised controlled trial with four recruitment sites in USA (4). Inclusion criteria included MDD diagnosis based on Structured Clinical Interview for DSM-IV (SCID), being in a current depressive episode of at least moderate severity defined as a 17-item Hamilton Depression Rating Scale (HAMD-17) score of a minimum of 14, and being medication-free. From EMBARC, EEG data were available in 281 MDD participants (185 women; mean age 37.18 years, SD 14.78 years) and 39 healthy control participants (24 women; mean age 37.21 years, SD 13.29 years), acquired at baseline before treatment initiation. In the initial treatment phase, MDD participants were randomised to receive either SSRI, sertraline, or placebo medication for 8 weeks. Treatment response was defined as achieving a ≥50% reduction in HAMD-17 score from baseline to week 8.

In EMBARC, resting-state EEG was collected at four clinical sites following a standardized acquisition protocol (4). EEG data were recorded in four 2-minute blocks at baseline, alternating between eyes-open and eyes-closed conditions. For consistency

with the CAN-BIND protocol, only the eyes-closed segments were included in the current analyses. Recordings were obtained using 61-channel EEG systems based on the standard 10–20 layout, referenced to linked mastoids, with a sampling rate of 500 Hz. Impedances were maintained below 10 kΩ, and the BrainVision system was used for data acquisition.

Summary of clinical features

Mean baseline depressive severity scores were CAN-BIND D1 (MADRS mean = 28.10, SD = 5.22), CAN-BIND D2 (MADRS mean = 30.18, SD = 5.45), EMBARC D1 (HAMD-17 mean = 19.42, SD = 3.82), and EMBARC D2 (HAMD-17 mean = 19.68, SD = 3.63). Following treatment: CAN-BIND D1 (MADRS mean = 17.53, SD = 5.36), CAN-BIND D2 (MADRS mean = 17.67, SD = 5.89), EMBARC D1 (HAMD-17 mean = 11.63, SD = 7.50) and EMBARC D2 (HAMD-17 mean = 11.50, SD = 6.29). In CAN-BIND, all MDD participants received open-label SSRI medication (escitalopram 10-20 mg, flexible dosage x 8 weeks). In EMBARC, participants were randomized to either SSRI medication (D1: 54 participants; D2: 33 participants) or placebo for 8 weeks (D1: 47 participants; D2: 49 participants). The SSRI administered was sertraline, with a flexible dose range of 50–200 mg/day, titrated over the first 3 weeks and maintained based on clinical response and tolerability. Treatment response was attained following the course of treatments in, D1: 50 MDD (34 women) and D2: 48 MDD (34 women), and persistent depressive symptoms were observed in, D1: 66 MDD (36 women) and D2: 73 MDD (51 women) (Table 2).

EEG Feature Extraction

Resting-state EEG features were derived from preprocessed, artifact-cleaned data using MATLAB (R2024b), with scripts adapted from the Sheffield Autism Biomarkers toolbox, which has been previously applied to large-scale clinical EEG datasets. All analyses were performed on eyes-closed resting-state EEG segments acquired at baseline, prior to any antidepressant or placebo administration.

Epoching and Filtering

Continuous EEG data were segmented into non-overlapping 2-second epochs. To minimise edge artifacts introduced by time–frequency decomposition, each epoch was mirrored by reflecting the signal at both temporal boundaries prior to filtering and convolution. EEG data were high-pass filtered at 0.5 Hz and low-pass filtered at 200 Hz using zero-phase finite impulse response filters implemented via MATLAB's highpass and lowpass functions. Line noise at 60 Hz was attenuated using a notch filter (designNotchPeakIIR with forward–backward filtering). Following time–frequency analysis, mirrored segments were discarded.

Spectral Power Estimation

Time–frequency decomposition was performed using complex Morlet wavelet convolution across 100 logarithmically spaced frequencies from 2 to 80 Hz. The resulting complex-valued output was converted to power by taking the squared magnitude. Power values were averaged across epochs, yielding a 32 (electrodes) × 100 (frequencies) power matrix for each participant.

For group-level analyses, spectral power was averaged within canonical frequency bands: delta (2–4 Hz), theta (4–8 Hz), alpha (8–14 Hz), beta (14–30 Hz), and gamma (30–80 Hz). Both absolute and relative power measures were computed. Absolute power values (μV^2) were $\log_{10}$-transformed to reduce positive skew, while relative power values were arcsine square-root transformed to stabilise variance prior to standardisation.

Frontal Alpha Asymmetry

Frontal alpha asymmetry (FAA) was computed using log-transformed alpha-band (8–14 Hz) power. Alpha power was first averaged across left frontal electrodes (F3, F7) and right frontal electrodes (F4, F8). FAA was calculated as the difference between right and left log-transformed alpha power values, such that more positive values indicate relatively greater right-hemisphere alpha activity.

Multiscale Sample Entropy

Multiscale sample entropy (MSE) was computed to quantify EEG signal complexity across multiple temporal scales. EEG signals were resampled to 1000 Hz and segmented into 2-second epochs. Coarse graining was performed by averaging non-overlapping windows of length τ, producing progressively smoothed time series for each scale. Twenty time scales were computed, capturing both fine- and coarse-grained temporal dynamics.

Sample entropy was calculated at each time scale using a pattern length of $m = 2$ and a similarity tolerance of $r = 0.30 \times$ standard deviation of the signal. Prior to entropy computation, all signals were z-scored to control for amplitude-related bias. The resulting MSE output consisted of a 32-channel × 20-scale matrix per participant, averaged across epochs.

Inter-Site Phase Clustering

Inter-site phase clustering (ISPC) was used to quantify phase synchrony between electrode pairs across the same 100 frequencies used for spectral power estimation. Prior to ISPC computation, signals were transformed using a surface Laplacian to enhance spatial specificity and reduce volume conduction effects.

ISPC was calculated as the consistency of phase differences between electrode pairs across time according to the formula:

$$\text{ISPC} = \frac{1}{n} \left| \sum_{t=1}^{n} e^{i(\phi_{x,t} - \phi_{y,t})} \right|$$

where $\phi_{x,t}$and $\phi_{y,t}$denote the instantaneous phase angles of electrodes x and y at time t, n is the number of time points, and i is the imaginary unit. ISPC values range from 0 (no phase consistency) to 1 (perfect phase locking). This procedure yielded a 32 × 32 × 100 ISPC matrix per participant, averaged across epochs.

Regional Grouping and Asymmetry Derivations

To reduce dimensionality and account for spatial correlation among electrodes, EEG features were summarised by averaging channels within anatomically coherent regional groupings. These included bilateral frontal, centro-parietal, occipito-parietal, lateral, and hemispheric composites, as well as midline regions.

Hemispheric asymmetry indices were computed using systematic electrode pairings. For power and MSE features, asymmetry was calculated as a normalised difference, (A − B) / (A + B), where A and B represent paired electrodes. For ISPC, which intrinsically reflects inter-electrode relationships, no transformation was applied. Electrode ordering was consistently maintained so that sign directionality reflected rostral–caudal or lateralised gradients.

Channel Grouping and Asymmetry Derivations

Because the analytical pipeline produced values for every electrode across a finely resolved frequency range, the dataset was extremely high-dimensional; for example, a simple power matrix of 32 electrodes by 100 frequencies would yield more than 3,000 variables. Adjacent electrodes and neighbouring frequency bins tend to be highly correlated and treating all these raw estimates as individual dependent variables would make the modelling intractable. The data were summarised by averaging channels within anatomically coherent regions and by organising the spectral estimates into canonical frequency bands. This resulted in thirteen regional groupings, each defined by conventional electrode sets. The right frontal region comprised FP2, AF4, F4, and F8, whereas the left frontal region comprised FP1, AF3, F3, and F7. Right centro-parietal activity was averaged from FC2, FC6, C4, CP2, and CP6, and the left counterpart from FC1, FC5, C3, CP1, and CP5. The right occipito-parietal region included P4, P8, PO4, and O2, and the left occipito-parietal region included P3, P7, PO3, and O1. A broader frontal composite included FP1, FP2, AF3, AF4, F3, F4, and FZ. The occipital composite covered PO3, PO4, O1, O2, and OZ, and the central grouping included FZ, CZ, PZ, and OZ. Additional lateralised groups comprised the left lateral electrodes F7, FC5, T7, CP5, and P7, as well as the right lateral electrodes F8, FC6, T8, CP6, and P8. Finally, whole-

hemisphere summaries were produced for the right hemisphere (FP2, AF4, F4, F8, FC6, FC2, T8, C4, CP6, CP2, P8, P4, PO4, O2) and the left hemisphere (FP1, AF3, F3, F7, FC5, FC1, T7, C3, CP5, CP1, P7, P3, PO3, O1).

Given the prominence of hemispheric asymmetry in the resting-state EEG literature, a set of asymmetry-sensitive measures was derived using systematic electrode pairings. Each asymmetry score was computed as a normalised difference, (A − B) / (A + B), except in the case of ISPC where the values already represent inter-electrode relationships and no transformation was needed. This framework captured several asymmetry dimensions. Interhemispheric contrasts were constructed using FP1–FP2, F3–F4, F7–F8, C3–C4, T7–T8, P3–P4, P7–P8, and O1–O2. Rostrocaudal gradients within the left hemisphere were constructed from the ordered pairings O1–P3, P3–C3, P7–T7, C3–F3, T7–F7, and CP1–FC1, while the corresponding right-hemisphere gradients used O2–P4, P4–C4, P8–T8, C4–F4, T8–F8, and CP2–FC2. Mediolateral gradients were also derived, using P7–P3, CP5–CP1, T7–C3, FC5–FC1, and F7–F3 on the left side, and P8–P4, CP6–CP2, T8–C4, FC6–FC2, and F8–F4 on the right side. Across all asymmetry comparisons, the ordering of electrodes was carefully maintained so that the first electrode in each pair consistently represented the more rostral or lateral site, ensuring that the sign and directional interpretation of each index remained stable across all configurations.

References

1. Kennedy, S. H., Lam, R. W., McIntyre, R. S., Tourjman, S. V., Bhat, V., Blier, P., … Milev, R. V. (2019). Canadian Network for Mood and Anxiety Treatments (CANMAT) 2016 clinical guidelines for the management of adults with major depressive disorder: Section 3. Pharmacological treatments. *Canadian Journal of Psychiatry, 64*(4), 287–303. https://doi.org/10.1177/0706743719859417

2. Farzan, F., Atluri, S., Frehlich, M., Dharsee, M., Waskow, I., Barr, M. S., … Daskalakis, Z. J. (2017). Standardization of electroencephalography for multi-site studies in major depression: A report from the CAN-BIND study. *Journal of Affective Disorders, 217*, 74–83. https://doi.org/10.1016/j.jad.2017.03.054

3. Lam, R. W., Milev, R., Rotzinger, S., Andreazza, A. C., Blier, P., Brenner, C., … Kennedy, S. H. (2016). Discovering biomarkers for antidepressant response: Protocol from the Canadian Biomarker Integration Network in Depression (CAN-BIND) and clinical characteristics of the first patient cohort. *BMC Psychiatry, 16*, 105. https://doi.org/10.1186/s12888-016-0785-x

4. Trivedi, M. H., McGrath, P. J., Fava, M., Parsey, R. V., Kurian, B. T., Phillips, M. L., … Weissman, M. M. (2016). Establishing moderators and biosignatures of antidepressant response in clinical care (EMBARC): Rationale and design.

Journal of Psychiatric Research, 78, 11–23. https://doi.org/10.1016/j.jpsychires.2016.03.001
